# Anti-interleukin-1 treatment among patients with familial Mediterranean fever resistant to colchicine treatment. Retrospective analysis

**DOI:** 10.1590/1516-3180.2018.0311101218

**Published:** 2019-05-08

**Authors:** Gokhan Sargin, Reyhan Kose, Taskin Senturk

**Affiliations:** I MD. Assistant Professor, Division of Rheumatology, Adnan Menderes Üniversitesi Tıp Fakültesi, Aydın, Turkey.; II MD. Fellowship-holder, Division of Rheumatology, Adnan Menderes Üniversitesi Tıp Fakültesi, Aydın, Turkey.; III MD. Professor, Division of Rheumatology, Adnan Menderes Üniversitesi Tıp Fakültesi, Aydın, Turkey.

**Keywords:** Familial Mediterranean fever, Quality of life, Interleukin-1 receptor antagonist protein, Colchicine

## Abstract

**BACKGROUND:**

Up to 5% of familial Mediterranean fever (FMF) cases are unresponsive to colchicine, through resistance, side effects and toxicity. Anakinra is an alternative treatment for FMF patients whose disease remains uncontrolled with colchicine. We aimed to evaluate anti-interleukin-1 treatment regarding clinical findings, laboratory parameters and quality of life (QoL) among FMF patients presenting resistance and toxicity towards colchicine.

**DESIGN AND SETTING:**

Descriptive observational study at the rheumatology clinic, Adnan Menderes University Medical School, Aydın, Turkey.

**METHODS:**

Among the patients included, age, sex, *MEFV* genotypes, acute-phase reactants, hepatic/renal function tests, average colchicine dose, disease duration, attack frequency, attack duration, disease severity, proteinuria, amyloidosis and QoL were evaluated. Colchicine resistance was defined as > 6 typical episodes/year or > 3 per 4-6 months. Kolmogorov-Smirnov, Friedman and two-way analysis of variance tests were used for statistical analyses.

**RESULTS:**

Between 2015 and 2017, 14 FMF patients receiving anakinra were enrolled. The mean colchicine dose was 1.7 ± 0.3 mg/day before use of anakinra. Ten patients were attack-free after treatment, while three showed reductions of at least 50% in attack frequency, attack duration and disease severity. Proteinuria levels in all patients with renal amyloidosis decreased after treatment. QoL among patients with renal amyloidosis differed significantly from QoL among non-amyloidosis patients. Mean visual analogue scale scores significantly improved in both groups after use of anakinra.

**CONCLUSIONS:**

Use of anakinra reduced attack frequency and proteinuria and acute-phase reactant levels, and improved QoL, with only a few uncomplicated side effects among colchicine-resistant or intolerant FMF patients. Injection-site reactions of severity insufficient to require discontinuation of treatment were seen.

## INTRODUCTION

Familial Mediterranean fever (FMF) is the most common hereditary periodic fever syndrome and characterized by recurrent fever and serositis. This disease shows autosomal recessive inheritance and it is frequently observed in the Middle East and Mediterranean basin, especially among Arabs, Armenians, Jews and Turks.^1^^,^^2^

In 90% of FMF patients, clinical manifestations start to occur before they reach their second decade of life. Attacks usually last from 12 hours to 3-4 days and are characterized by fever, abdominal pain, chest pain, arthritis and erysipelas-like skin lesions. Amyloidosis is the most frightening complication because of its morbidity and mortality. The incidence of FMF-associated amyloidosis has been reduced through decreasing the delay in diagnosing this condition and through early treatment with colchicine.^1^^,^^2^^,^^3^

Colchicine is used both in preventing attacks and in treating amyloidosis. It is recommended that each FMF patient should receive continuous appropriate doses of colchicine.^4^ However, up to 5% of these patients are unresponsive to colchicine because of side effects, tolerability problems and colchicine resistance.^5^ Certain pyrin mutations and polymorphism of the drug transporter gene adenosine triphosphate-binding cassette subfamily B member 1 (ABCB1) may cause colchicine resistance. However, the exact mechanism remains unknown.^5^^,^^6^

The efficacy of serotonin reuptake inhibitors, interferon-alpha, thalidomide, azathioprine, anti-tumor necrosis factor agents and drugs that block interleukin-1, such as anakinra, canakinumab and rilonacept, has been investigated among colchicine-intolerant or resistant patients.^7^^,^^8^^,^^9^^,^^10^^,^^11^^,^^12^ Anakinra is a recombinant non-glycosylated homologous human interleukin-1 receptor antagonist that competitively binds to interleukin-1α and interleukin-1β, which are interleukin-1 receptors. Studies on FMF patients taking anakinra have demonstrated that use of this drug leads to reduction in the frequency of attacks and in acute-phase reactant levels. Moreover, use of anakinra has been reported to reduce proteinuria levels.^13^^,^^14^^,^^15^^,^^16^^,^^17^ Additionally, successful treatment of gout, chronic kidney disease and aplastic anemia through use of anakinra has been reported.^18^

## OBJECTIVE

The aim of this study was to evaluate clinical parameters such as severity of illness, attack duration, attack frequency and presence of amyloidosis among FMF patients presenting resistance and toxicity towards colchicine. We also evaluated acute-phase reactants, proteinuria, side effects, genetic mutations and quality of life among patients with and without amyloidosis, in relation to treatment with anakinra.

## METHODS

This was a single-center retrospective descriptive study, in which patients receiving anakinra between 2015 and 2017 were enrolled. These patients had been admitted to the rheumatology clinic of Adnan Menderes University Medical School, in Aydın, Turkey, with a diagnosis of FMF. They had been treated with anakinra (100 mg/day, subcutaneously) because they had presented side effects, intolerance or resistance to colchicine. Patients with histories of infection, malignancy or other autoinflammatory diseases were not included in the study.

A signed informed consent form was obtained from each patient for whom anakinra treatment was planned. Ethics committee approval was received for this study, from the ethics committee of our university (approval number: 2017/1258; approval date: November 9, 2017).

The Tel-Hashomer criteria were used to make the diagnosis of FMF.^19^ The major criteria comprise presence of fever episodes accompanied by arthritis and/or serositis, AA-type amyloidosis without any predisposing disease and good response to colchicine. The minor criteria comprise presence of erysipelas-like erythema, occurrence of FMF in a first-degree relative and recurrence of fever attacks. A definitive diagnosis is made in the presence of two major or one major and two minor criteria. A probable diagnosis is made in the presence of one major criterion and one minor criterion.

Colchicine resistance was defined as more than six typical episodes per year or more than 3 episodes per 4-6 months.^3^

The relationship between quality of life and amyloidosis was evaluated, since amyloidosis is the most important complication that leads to morbidity and mortality among FMF patients. Qualityof life was assessed using a visual analogue scale (VAS) from 0 to 10 cm that represented, respectively, from poor to good quality of life.

Clinical and laboratory data were obtained from the patients’ records, retrospectively. Age, sex, *MEFV* genotypes, acute-phase reactants [erythrocyte sedimentation rate (ESR) and C-reactive protein (CRP)], hepatic/renal function tests, average colchicine dose, disease duration, attack frequency, attack duration, disease severity, proteinuria and amyloidosis were evaluated. Also,side effects that limit colchicine use, such as hepatic, neuromuscular and hematological toxicities, including rhabdomyolysis, aplastic anemia leukopenia, neutropenia and thrombocytopenia, were evaluated.

The normal reference ranges for ESR, CRP and 24-hour urinary protein excretion are as follows: 0-20 mm/h, 0-5 mg/dl and <150 mg. Renal biopsies were performed in cases of suspected renal amyloidosis. The results from 24-hour urinary protein excretion among the patients with renal amyloidosis, and their ESR and CRP levels, were evaluated and recorded at each visit.

The quantitative data were examined using the Kolmogorov-Smirnov test to ascertain whether they conformed to normal distribution. Descriptive statistics were presented as means ± standard deviations or medians and interquartile ranges for continuous variables and as frequencies and percentages for categorical variables. Wilcoxon’s and Friedman’s tests were used as non-parametric tests, while two-way analysis of variance (ANOVA) was used for repeated measurements. P < 0.05 was accepted as statistically significant. The Statistical Package for the Social Sciences, version 17.0 for Windows (SPSS Inc., Chicago, IL, USA), was used to analyze the data.

## RESULTS

Between 2015 and 2017, 14 patients (9 males and 5 females) receiving anakinra were enrolled. Most of the FMF patients included in this study were male and the mean age for all the FMF patients was 41.3 ± 10.7. The length of time for which they had had the diagnosis of FMF was 11.8 ± 8.8 years. The mean length of time that elapsed until the first follow-up visit was 11.4± 2.2weeks, and it was 26.4 ± 4.7 weeks until the second visit and 47.5 ± 5.0 weeks until the third visit.

All of the patients were taking colchicine regularly. Among these patients with a diagnosis of FMF, 50% presented renal amyloidosis, 14.3% had ankylosing spondylitis and 7.1% had adult-onset Still’s disease. The demographic and clinical features of the patients are summarized in Table 1.

**Table 1.** Demographic and clinical features of the patients with familial Mediterranean fever

	Total (n = 14)
Age (years), mean ± standard deviation	41.3 ± 10.7
Male, n Female, n	9 5
Colchicine dose (mg/day), mean ± standard deviation	1.7 ± 0.3
Disease duration (years), mean ± standard deviation	11.8
Fever, n (%)	14 (100%)
Serositis, n (%)	10 (71.4%)
Arthritis, n (%)	8 (57.1%)
Febrile myalgia, n (%)	1 (7.1%)
Chronic renal disease, n (%)	3 (21.4%)
Amyloidosis, n (%)	7 (50%)
Sacroiliitis, n (%)	2 (14.3%)

Colchicine resistance was present in 13 of these patients. Use of anakinra was started among these patients because of their intolerance of colchicine treatment and the toxic hepatitis that it led to. The mean colchicine dose was 1.7 ± 0.3mg/day in the beginning and the mean duration of anakinra treatment was 16.2 ± 8.9 months. There were no attacks after treatment in 10 patients, while the remaining three patients had a reduction in attack frequency of at least 50%. Moreover, these same three patients presented decreases in the severity and duration of the disease of more than 50%. We were unable to evaluate the response in one patient who received anakinra because this patient died due to amyloidosis and renal failure that was unrelated to use of anakinra, on the 10^th^ day of the therapy.

The mean age of the seven patients with renal amyloidosis (female-to-male ratio of 6:1) was 42 ± 10.9 years. The mean duration of their diagnosis of amyloidosis was 7.2 ± 5.5 years and their average colchicine dose was 1.8 ± 0.2 mg/day.

Genetic analysis results were available for 92.8% of all the FMF patients (13/14). The most frequently detected mutation was *M694V* (78.5%). The *MEFV* genotype of the patients with renal amyloidosis was *M694V* homozygote in three patients and there was one patient with each of the following: *E148Q* heterozygous/*M694V* heterozygous; *M694V* heterozygous/*R202Q* heterozygous; *M694V* homozygous/*R202Q* homozygous; and *R202Q* heterozygous mutation.

An injection-site reaction was observed in two patients. Neither of these was a serious reaction that would have required discontinuation of use of anakinra, and the reaction was controlled in both cases using topical corticosteroids and antihistaminic drugs. The treatment was halted in one of these two patients due to local injection-site infection and abscess development.

At the time of the first visit, a decrease in proteinuria levels was observed in four patients with amyloidosis, compared with the baseline. However, this did not reach statistical significance. A decrease in 24-hour urinary protein was observed in relation to all the patients with amyloidosis at the time of the third visit, compared with the pre-treatment values (Table 2).

**Table 2.** 24-hour urinary protein (mg) in familial Mediterranean fever patients with renal amyloidosis at baseline and at first, second and third visits

	24-hour urinary protein (mg)*
Baseline	13,995 (5,298.2-17,795)
First visit	5,875.1 (2,777.1-9,627)
Second visit	4,507 (2,596.8-10,197.3)
Third visit	2,508 (1,387.5-9,009.7)**

*Median (with interquartile range); **P = 0.02 (from analysis of variance, ANOVA), compared with before the treatment.

The ESR and CRP levels of the FMF patients were higher before anakinra treatment. The median ESR was 65.5 mm/h (range: 24-93) at the first visit relating to the treatment with anakinra and 25 mm/h (range: 13.7-58) at the second visit. Thelevels of the acute-phase reactants diminished over the course of the patients’ follow-up. There were significant differences in both ESR and CRP levels, compared with the pre-treatment situation. The ESR and CRP levels among the FMF patients at the baseline and at the first, second and third visits are shown in Table 3.

**Table 3.** Erythrocyte sedimentation rate and C-reactive protein (CRP) levels in familial Mediterranean fever patients at baseline and at first, second and third visits

Erythrocyte sedimentation rate (mm/h)	Median (with interquartile range)	P-value
Baseline	65.5 (24-93)	
First visit	28 (10.5-63.5)*	0.002
Second visit	25 (13.7-58)*	0.005
Third visit	15 (12-38)*	0.008
C-reactive protein (mg/dl)	Median (with interquartile range)	P-value
Baseline	41.9 (10.2-102.3)	
First visit	9.9 (3.1-24,1)*	0.002
Second visit	4.5 (1.4-10.8)*	0.005
Third visit	1.9 (0.8-17.3)*	0.02

*P < 0.05 (from analysis of variance, ANOVA) for the rates at the first, second and third visits, compared with baseline.

Furthermore, the quality of life score from the 10-cm VAS improved through use of anakinra, as observed at the first, second and third visits. The changes in VAS score among the FMF patients with and without amyloidosis are shown in Table 4.

**Table 4.** Changes to visual analogue scale scores among familial Mediterranean fever patients with and without amyloidosis at baseline and at first, second and third visits

Visual analogue scale (mean ± SD)	With amyloidosis	Without amyloidosis	P-value
Baseline	2.1 ± 1.0	4.8 ± 0.7	0.001
First visit	5.8 ± 0.9*	6.8 ± 0.7**	0.03
Second visit	8.0 ± 1.2*	9.0 ± 0.7**	0.1
Third visit	8.5 ± 1.0*	9.0 ± 0.8	0.1

*P < 0.05 (from analysis of variance, ANOVA) for visual analogue scale scores at the first, second and third visits, compared with baseline (with amyloidosis); **P<0.05 (from ANOVA) for visual analogue scale scores at the first and second visits compared with baseline (without amyloidosis).

## DISCUSSION

In this study, we evaluated 14 patients with FMF who were treated with anakinra because of side effects, toxicity or resistance caused by colchicine. It was found at the third visit that the proteinuria levels in all the renal amyloidosis patients had decreased.

Azathioprine, anti-tumor necrosis factor agents, thalidomide, interferon-alpha, serotonin reuptake inhibitors, anakinra, rilonacept and canakinumab can be considered to be alternative treatment options for refractory FMF.^7^^,^^8^^,^^9^^,^^10^^,^^11^^,^^12^ Because of FMF-associated mutations, inhibition of interleukin-1β and activation of nuclear factor kappa B are among the therapeutic aims of treatment targeted on interleukin-1.

Anakinra appears to be an alternative agent for treating colchicine-resistant or intolerant patients, with effects that have been reported in case reports, case series and some prospective studies.^12^^,^^13^^,^^14^^,^^15^^,^^16^^,^^17^ In two recent cohorts, anakinra and canakinumab were shown to be effective for decreasing attack frequency, serum acute-phase reactants and proteinuria among refractory FMF patients.^17^^,^^20^ Moreover, anakinra has been reported to be an effective and safe treatment among pregnant women with FMF.^21^ In our study, there were no cases of use of anakinra during pregnancy among women with FMF.

Mutations such as *E148Q, M680I, M694V, M694I* and *V726A* have been shown to be responsible for the cases of more than 80% of FMF patients.^2^ In our study, M694V was the most common mutation (78.5%).

AA-type secondary amyloidosis is typically seen in FMF cases. In a study conducted by the Turkish FMF study group, the frequency of amyloidosis among FMF patients was reported to be 12.9%.^22^ In our clinic, this rate is 3.4%.

It has been reported that interleukin-1 inhibitorsreduce proteinuria and stabilize or preserve renal functions over short-term follow-up.^23^ In our study, decreased 24-hour urinary protein levels in all patients with amyloidosis were observed at the time of the third visit, compared with the pre-treatment situation (Table 2).

Colchicine is effective for preventing FMF attacks, and also for diminishing the development of amyloidosis.^24^^,^^25^ It has been suggested that colchicine resistance is associated with mutation penetration in the *MEFV* gene, most frequently in individuals with the *M694V* homozygous mutation.^26^^,^^27^ All of the patients included in the present study were using colchicine regularly. Colchicine resistance was present in 13 of these patients. In one patient, use of anakinra was started due to intolerance and toxic hepatitis. Themean colchicine dose was 1.7 ± 0.3 mg/day in the beginning. It was higher (1.8 ± 0.2 mg/day) among patients with amyloidosis than among those without amyloidosis. The median attack frequency was 8 (range: 6-8) among our patients, over the last six months before anakinra treatment.

In a randomized, double-blind, placebo-controlled study, a total of 25 patients were randomized such that 12 received anakinra and 13 received placebo.^28^ It was found that the frequency of attacks was significantly lower in the anakinra group than in the placebo group, particularly regarding joint attacks.^28^ We found that there was a complete response to anakinra in 10 patients, in terms of attack frequency, while the remaining three patients presented improvements of at least 50%. Moreover, these three patients presented decreases in the severity and duration of the disease of more than 50%.

FMF affects the quality of life adversely. It has been reported that the quality of life among FMF patients is lower than that ofhealthy controls.^29^ Inverse correlations between the number of attacks, disease severity and quality of life have been found, without any difference between the sexes.^29^ Depression and anxiety are also common among FMF patients, compared with healthy individuals.^30^ Quality of life has been shown to be better among patients who received anakinra, compared with a placebo group, but without any significant difference in adverse events between the anakinra group (mean ± SD: 2.03 ± 1.75) and the placebo group (mean ± SD: 3.34 ± 2.5).^28^

The relationship between quality of life, as assessed using the Short Form-36 (SF-36) questionnaire, and disease parameters has also been investigated. It has been shown that FMF affected both mental and general health parameters, and also that it influenced the physical/physical role/emotional role functions.^31^

We used a VAS to assess quality of life. The mean VAS score of all the patients before use of anakinra was 3.5 ± 1.6 cm, and it became significantly higher among the FMF patients who were treated with anakinra. There was a significant difference in quality of life between the patients with renal amyloidosis (2.1 ± 1.0 cm) and those in the non-amyloidosis group (4.8 ± 0.7 cm) before the treatment (P = 0.001). At the time of the first visit, the mean VAS scores of both of these groups with use of anakinra were found to have increased significantly, compared with the pre-treatment situation.

The limitations of our study were that its nature was descriptive and observational, without any control group or randomization. Furthermore, we did not use SF-36 to evaluate the quality of life.

The highlight of our study was that it provided some evidence that use of anakinra reduced the attack frequency and proteinuria and acute-phase reactant levels, and that it improved quality of life. Consequently, although colchicine remains the first choice for treating FMF, it can be confirmed that anakinra is an effective and safe alternative treatment for colchicine-resistant patients. Use of anakinra decreased the frequency of attacks and led to decreased amounts of proteinuria in cases of renal amyloidosis. It was also found that use of anakinra led to improvement in quality of life.

## CONCLUSIONS

Anakinra reduced attack frequency and proteinuria and acute-phase reactant levels and improved quality of life, with only a few uncomplicated side effects in most colchicine-resistant or intolerant FMF patients. Injection-site reactions were seen but were not severe enough to require discontinuation of treatment.
